# Cellular Senescence-Related Genes: Predicting Prognosis in Gastric Cancer

**DOI:** 10.3389/fgene.2022.909546

**Published:** 2022-06-01

**Authors:** Longfei Dai, Xu Wang, Tao Bai, Jianjun Liu, Bo Chen, Wenqi Yang

**Affiliations:** Department of General Surgery, The First Affiliated Hospital of Anhui Medical University, Hefei, China

**Keywords:** cellular senescence, GC, prognosis, chemotherapy, immunotherapy

## Abstract

Our study aimed to explore the effect of cellular senescence and to find potential therapeutic strategies for gastric cancer. Cellular senescence-related genes were acquired from the CellAge database, while gastric cancer data were obtained from GEO and TCGA databases. SMARCA4 had the highest mutation frequency (6%), and it was linked to higher overall survival (OS) and progression-free survival (PFS). The gastric cancer data in TCGA database served as a training set to construct a prognostic risk score signature, and GEO data were used as a testing set to validate the accuracy of the signature. Patients with the low-risk score group had a longer survival time, while the high-risk score group is the opposite. Patients with low-risk scores had higher immune infiltration and active immune-related pathways. The results of drug sensitivity analysis and the TIDE algorithm showed that the low-risk score group was more susceptible to chemotherapy and immunotherapy. Most patients with mutation genes had a lower risk score than the wild type. Therefore, the risk score signature with cellular senescence-related genes can predict gastric cancer prognosis and identify gastric cancer patients who are sensitive to chemotherapy and immunotherapy.

## Introduction

Nowadays, cancer is the primary cause of threat to human health (Bray et al., 2021). It ranked fifth in incidence and fourth in mortality worldwide, while the number of GC diagnosed in 2020 was more than 1 million and the number of deaths was more than 700,000 (Sung et al., 2021). There are various treatments for GC, such as surgery, radiotherapy, targeted therapy, and immunotherapy (Joshi and Badgwell, 2021). Currently, early-stage GC is mainly treated by surgical resection (Eusebi et al., 2020). It was found that early-stage GC treated by surgery has a 5-year survival probability above 60%, but late-stage GC is only between 18% and 50% (Sexton et al., 2020). Moreover, the appearance of resistance to chemotherapy drugs has greatly reduced the effectiveness of chemotherapy (Zhang et al., 2022). Therefore, a new therapeutic strategy is urgently needed to improve this situation.

Cellular senescence is an irreversible way of cell proliferation cessation. It not only stops the cell division cycle but also activates the senescence-associated secretory phenotype (SASP), which affects the cellular metabolism (Birch and Gil, 2020). Cellular senescence is a Jekyll and Hyde phenomenon, that is, both beneficial in inhibiting the division of DNA-damaged cells to form tumors and deleterious due to the promotion of cancer cell invasion and distant metastasis, especially in cells with stronger SASP (Coppé et al., 2008; Demirci et al., 2021; Yasuda et al., 2021). Studying the effect of cellular senescence in GC could help develop a new approach to cancer therapy (Zhou et al., 2022). Therefore, the study of cellular senescence in GC is crucial.

Machine-learning-derived signatures are useful in predicting cancer prognosis and guiding immunotherapy (Liu Z et al., 2021; Liu et al., 2022a; Liu et al., 2022b). In the study, we constructed a cellular senescence prognostic risk score signature by analyzing the role of cellular senescence in GC. The signature can independently predict GC patients’ prognosis and effectively differentiate patients who are more sensitive to chemotherapy and immunotherapy. The findings of this study may provide new strategies for exploring the therapy of GC.

## Methods

### Acquisition of Gastric Cancer Samples and Cellular Senescence-Related Genes

The process diagram is shown in Supplementary Figure S1. We acquired transcriptome data, clinical information, and mutation information of GC from TCGA databases. The gene symbol ID was translated to gene name in transcriptome data. Tumor mutation load (TMB) was calculated. TMB refers to how many bases per million bases are mutated. The platform file (GPL6947) and probe matrix file (GSE84437) were extracted from GEO. The correspondence between the probe matrix and gene names was found according to the platform file annotation information. The probe matrix was converted to a gene matrix to obtain the expression of each gene. Cellular senescence-related genes were downloaded from CellAge. A total of 279 cellular senescence-related genes were included in this study (Supplementary Table S1).

### Identification of Prognostic Differential Genes

Differential analysis was conducted by the “limma” package to select differentially expressed genes (DEGs) in normal samples and tumor samples. DEGs were visualized by drawing heat maps and volcano maps. Next, we also extracted the expression of DEGs. Expression data and survival data were merged. Prognostic-associated genes were identified based on univariate Cox analysis. The waterfall plot of prognostic genes was plotted by the “maftools” package to obtain the mutation frequency of each gene.

### Constructing and Validating a Prognostic Signature

TCGA data were used as a training set to construct the prognostic model, and GEO data served as a testing set to validate the model accuracy. Formula: riskscore = ∑i1(Coefi∗ExpGenei). “Coef,” regression coefficient; “ExpGene,” gene expression. The risk score was acquired for each sample based on the model formula. Training and testing sets were separated into two groups of high and low risk according to the median risk score. Principal component analysis (PCA) was performed to demonstrate the accuracy of distinguishing the two groups based on the signature. The survival difference in the two groups was compared by Kaplan–Meier analysis. The predictive accuracy of the signature was evaluated by plotting ROC curves using the “survivalROC” package. The signature was explored as an independent prognostic factor by univariate and multivariate Cox analyses. The “ggpubr” package was employed to investigate the differences in risk scores among clinical features. Immunotyping analysis was conducted to explore whether risk scores were different among different immunotypes.

### Development of a Nomogram

By using “regplot” and “rms” packages, nomogram and calibration curves were developed. Total points were obtained based on summing the scores of the clinical characteristics in the nomogram to predict patients’ survival. We also used the “timeROC” package to draw ROC curves to compare the accuracy of the nomogram and clinical characteristics in predicting survival. Then, we confirmed whether the nomogram could be used as an independent predictor of prognosis based on univariate and multivariate Cox analyses. C-index curves were constructed using the “survcomp” package.

### Exploring the Association Between Risk Scores and Immunotherapy

Immune cell infiltration analysis was undertaken to acquire the immune cell content of each sample (Supplementary Table S2). “reshape2” and “ggpubr” packages were performed to observe immune cell differences and immune-related functional differences between different risk groups. The “GSVA” package was applied to explore the functional or pathway differences between different risk groups. We acquired the reference gene set “c2.cp.kegg.v7.1.symbols” from the Molecular Signature Database (https://www.gsea-msigdb.org/gsea/msigdb). The samples were categorized into mutation and wild type based on the gene mutation status. The difference in risk scores between mutation and wild type was observed by plotting box plots with the “ggpubr” package. Drug sensitivity analysis was conducted by the pRRophetic package (https://www.cancerrxgene.org/) to investigate IC_50_ (the half-maximal inhibitory concentration) differences between high- and low-risk groups. We also used the TIDE (http://tide.dfci.harvard.edu/) algorithm to predict the response of different risk groups to immunotherapy.

### Enrichment Analysis of Differentially Expressed Genes

The expression differences of cellular senescence-related genes in tumor and normal samples were further analyzed by the “limma” package. We also conducted GO and KEGG enrichment analyses for DEGs with the “clusterProfiler” package.

### Construction of the Protein–Protein Interaction Network

A PPI network (interaction score >0.70) was constructed using the STRING database (https://string-db.org/). PPI network data were further processed using Cytoscape software (https://cytoscape.org/). The plugin cytoHubba was applied to explore the hub genes of DEGs. “limma” and “beeswarm” packages were used to investigate the differentially expressed hub genes in normal and tumor tissues. The samples were categorized into high- and low-expression groups based on the median expression values of the hub genes. Survival differences between the two groups were investigated by Kaplan–Meier analysis. Finally, we also explored the differences in the gene expression in immune infiltration and different clinical features.

### Statistical Analysis

R 4.1.2 and Strawberry-Perl-5.32.1.1 were employed in this study. *p*-values less than 0.05 were regarded as statistically significant. Survival differences between different groups were investigated by performing a Kaplan–Meier analysis. The independent predictors of GC were identified by univariate and multivariate Cox analyses. The accuracy of the signature and nomogram in predicting survival was explored by ROC analysis.

## Results

### Identification of Cellular Senescence-Related Differential Genes

**FIGURE 1:**
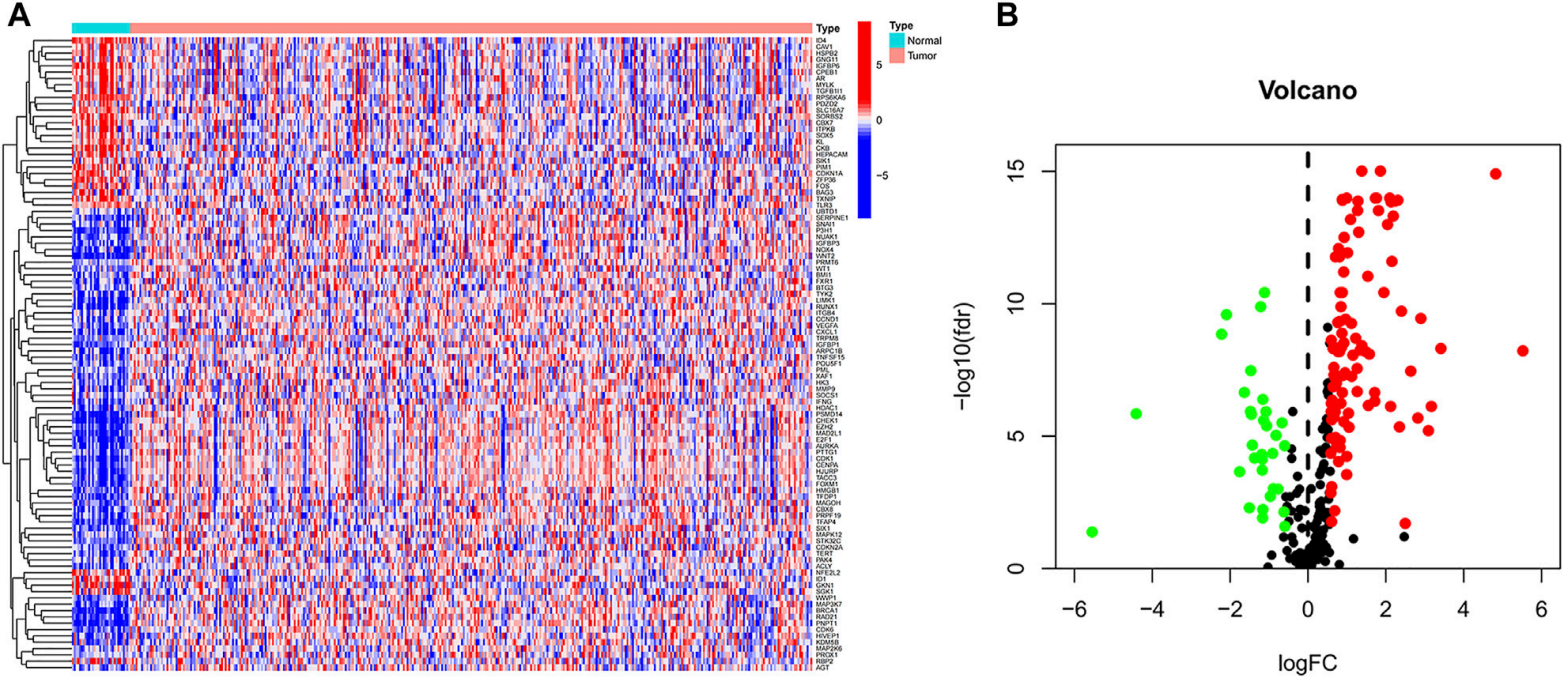
Cellular senescence-related differential genes between tumor samples and normal samples. **(A)** Heat map. **(B)** Volcano plot. Green, downregulated; red, upregulated.

In TCGA data, we identified 135 differential genes by comparing the difference in the expression of cellular senescence-related genes in tumor and normal tissue samples (FDR <0.05 and logFC = 0.585). The heat map (Figure 1A) and volcano map (Figure 1B) visualized the aforementioned results. There were 32 genes significantly hyper-expressed and 103 genes significantly down-expressed in the tumor tissue samples.

### Construction of a Prognostic Signature

**FIGURE 2:**
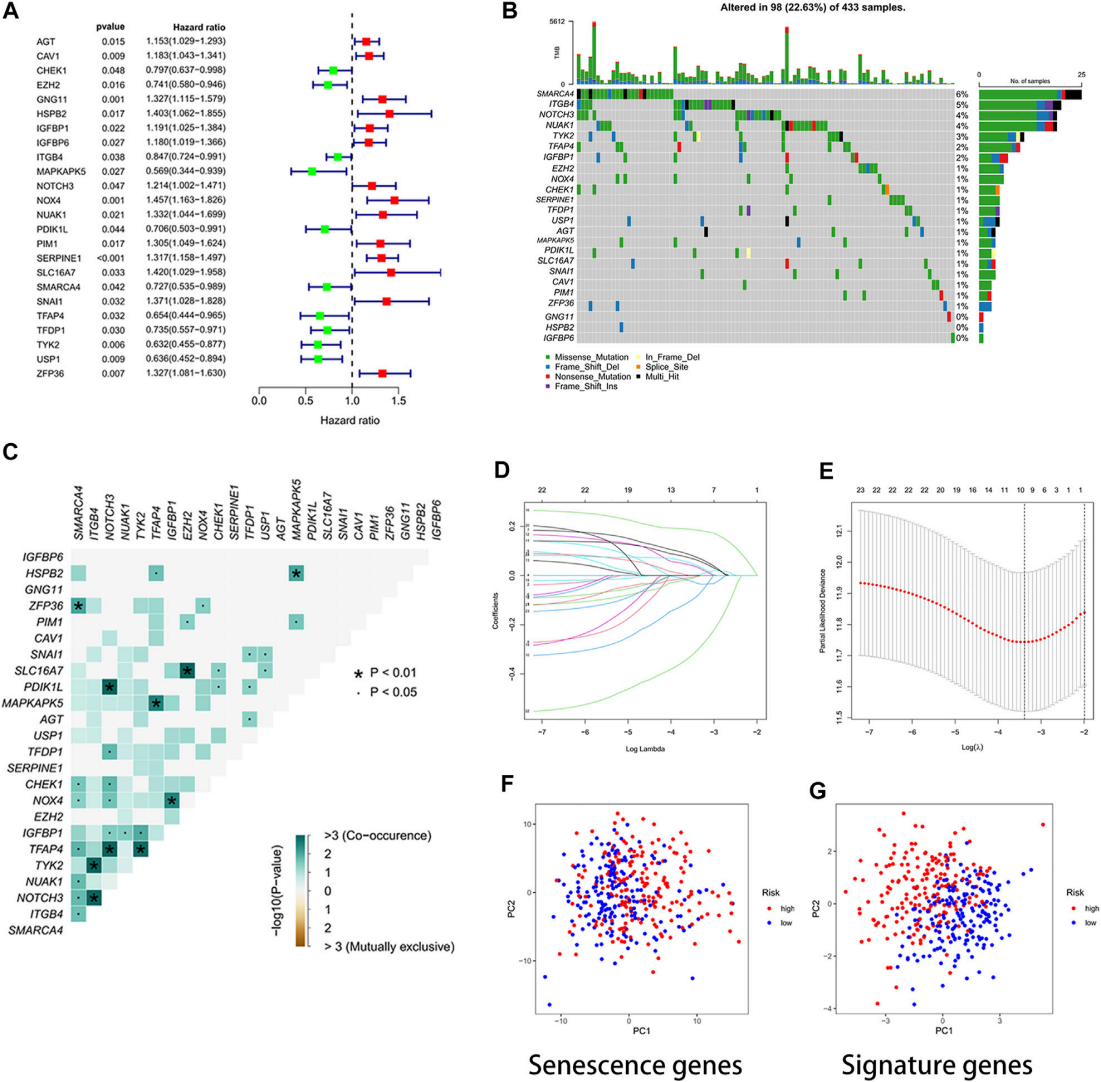
Developing a prognostic signature. **(A)** Forest plot. The 24 cellular senescence-related genes associated with GC prognosis. **(B)** Waterfall plot. Mutation frequency of 24 cellular senescence-associated genes. A total of 98 sample mutations have occurred in 433 gastric cancer samples. **(C)** Mutation co-occurrence and exclusion analysis. Green, co-occurrence; purple, exclusion. **(D)** LASSO regression coefficients. **(E)** Identified genes were used to construct a signature. **(F)** PCA diagram. The high- and low-risk groups were distinguished by cellular senescence-associated genes. Groups marked in blue represent low-risk patients, and groups marked in red represent high-risk patients. **(G)** PCA plot. The risk score signature genes distinguished high- and low-risk groups of patients with high accuracy.

The 24 cellular senescence DEGs associated with GC prognosis were identified through univariate Cox analysis, such as SMARCA4 (Figure 2A). Figure 2B shows the somatic mutations of 24 genes with a mutation frequency of 22.63% (98 out of 433 GC samples showed mutations in cellular senescence-related genes). Of these, SMARCA4 had the highest mutation frequency (6%), while GNG11 and IGFBP6 were not mutated (0%). We also found a significant difference between the high- and low-expression of SMARCA4, and patients with high expression of SMARCA4 were associated with higher overall survival (OS) and progression-free survival (PFS) (Supplementary Figure S2). Interestingly, there was a mutation co-occurrence relationship between SMARCA4 and ZFP36, ITGB4 and TYK2, ITGB4 and NOTCH3, NOTCH3 and PDIK1L, NOTCH3 and TFAP4, TFAP4 and MAPKAPK5, TFAP4 and TYK2, EZH2 and SLC16A7, IGFBP1 and NOX4, and HSPB2 and MAPKAPK5 (Figure 2C). Next, we further identified 24 cellular senescence DEGs associated with gastric cancer prognosis by using the least absolute shrinkage and selection operator (LASSO) Cox regression analysis. A total of 11 genes (AGT, CHEK1, GNG11, IGFBP1, MAPKAPK5, NOX4, SERPINE1, TFDP1, TYK2, USP1, and ZFP36) were identified (Figures 2D,E). Meanwhile, we developed a prognostic risk score signature based on the 11 genes mentioned earlier in the training set (Supplementary Table S3). Formula: risk score = (0.0617318899456387) × AGT + (−0.004416679732613) × CHEK1 + (0.00146582976956934) × GNG11 + (0.027549739978882) × IGFBP1 + (−0.0823743685561005) × MAPKAPK5 + (0.0337754127670565) × NOX4 + (0.184215619451523) × SERPINE1 + (−0.00197579186740112) × TFDP1+ (−0.303214268137102) × TYK2 + (−0.0314400326211636) × USP1 + (0.0400501256934474) × ZFP36 (Supplementary Table S3). We found that the signature could accurately distinguish low-risk and high-risk samples in GC by PCA (Figures 2F,G).

### Validation of Signature Genes in the HPA Database

To investigate the protein expression of the signature genes in normal and gastric cancer tissues, we downloaded immunohistochemical images of gastric cancer tissues and normal tissues from the Human Protein Atlas database (https://www.proteinatlas.org/). We found that MAPKAPK5 and USP1 proteins were highly expressed in gastric cancer tissues, while the ZFP36 protein was lowly expressed in tumor tissues (Supplementary Figure S3).

### Predicting Survival With the Risk Score Signature

**FIGURE 3:**
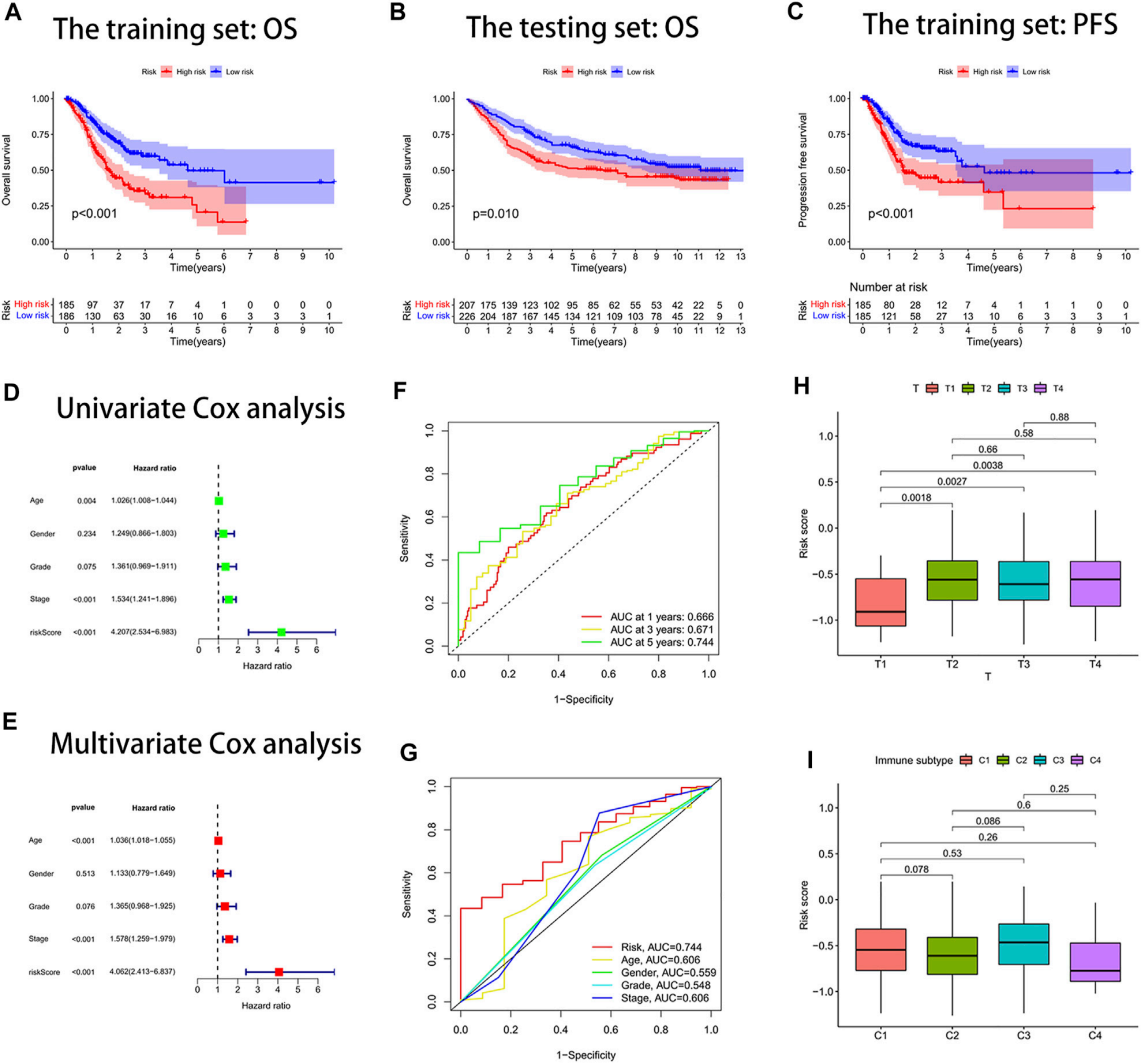
Risk score signature predicted prognosis for gastric cancer patients. **(A)** Overall survival (OS) curves of the high- and low-risk groups in the training set. **(B)** Overall survival (OS) curves of the high- and low-risk groups in the testing set. **(C)** Progression-free survival (PFS) curves of the high- and low-risk groups in the training set. **(D)** Univariate Cox analysis. **(E)** Multivariate Cox analysis. **(F)** Area under the ROC curve (AUC) for the risk score signature that predicted 1-year, 3-year, and 5-year overall survival. **(G)** ROC curves. The area under the ROC curve (AUC) for the risk score was the highest at 0.744. **(H)** Box plot of the difference in risk score for patients with different T-stages. **(I)** Box line plot of the difference in the risk score for patients with different immune subtypes.

Through survival curves, we observed longer overall survival (OS) and progression-free survival (PFS) in the low-risk subgroup of the training set (Figures 3A,C). The aforementioned results were confirmed in the testing set (Figure 3B). The results of univariate and multivariate Cox analyses indicated that the risk score signature could be used as an independent prognostic factor for gastric cancer patients independently of other clinical characteristics (Figures 3D,E). The signature was very accurate in predicting survival in patients with gastric cancer, with an area under the ROC curve (AUC) of more than 0.60 for predicting 1-, 3-, and 5-year survival (Figure 3F). We found the largest area under the ROC curve for the risk score (AUC = 0.744), which indicated that the signature predicted survival better than other clinical characteristics (Figure 3G). We further investigated whether there were differences in risk scores across clinical characteristics (age, gender, grade, stage, and TNM stage). We found an increased risk for patients after the T1 stage and no significant change in risk for patients after the T2 stage (Figure 3H). In contrast, there were no significant differences in risk scores for other clinical characteristics (Supplementary Figure S4). Interestingly, we also found no difference in risk scores for immune subtypes (Figure 3I).

### Development of a Nomogram

**FIGURE 4:**
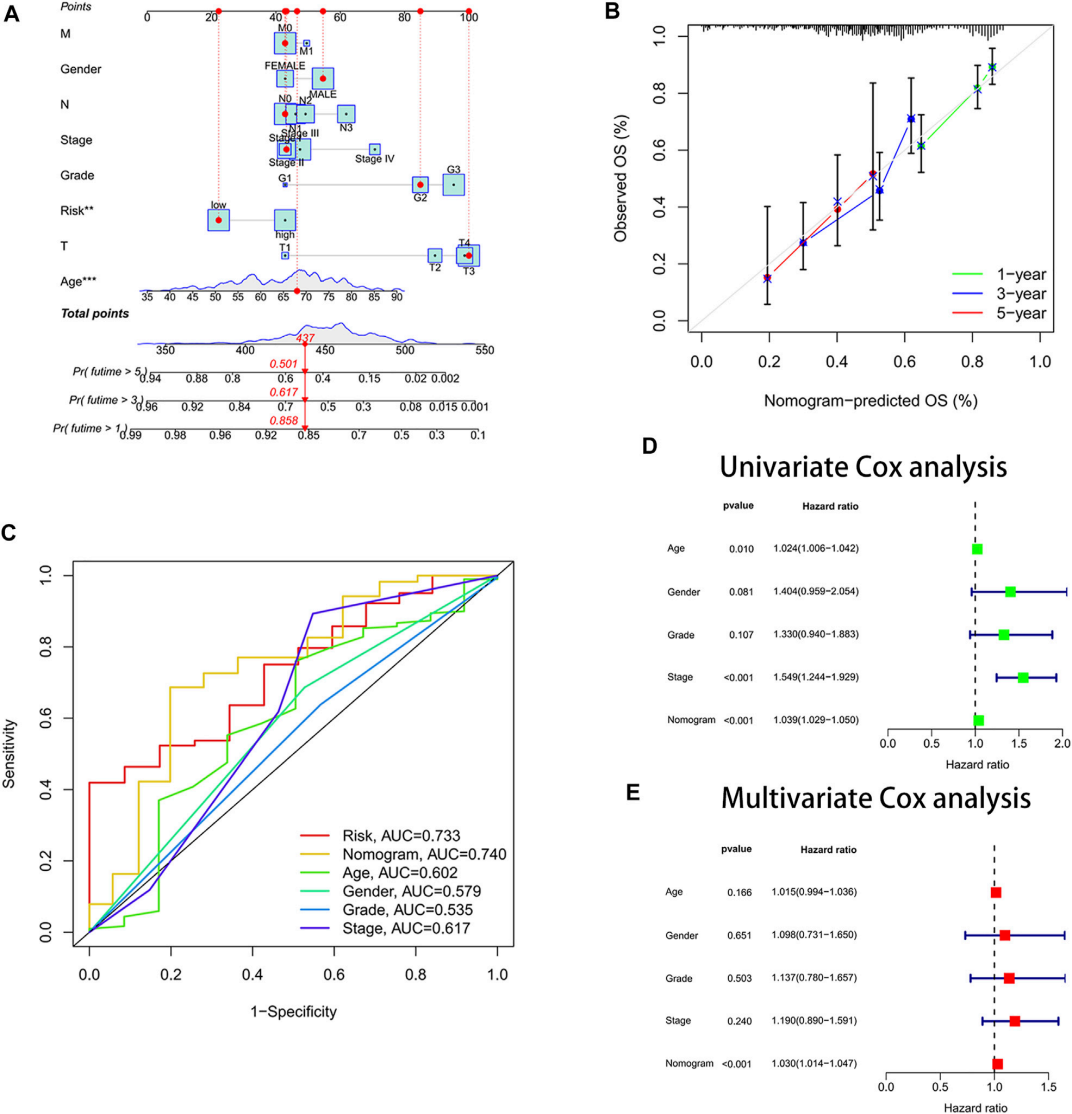
Constructed a nomogram for predicting survival. **(A)** Nomogram. **(B)** Calibration curves. The y-axis is the actual survival rate, and the x-axis is the predicted survival rate. **(C)** Area under the ROC curve (AUC) of the nomogram. **(D)** Univariate Cox analysis. **(E)** Multivariate Cox analysis.

We drew a nomogram to predict patients’ survival (Figure 4A). When patients’ total point was 437, the predicted survival rate at 1-year was more than 0.858, the predicted survival rate at 3-year was more than 0.617, and the predicted survival rate at 5-year was more than 0.501. We found that the actual survival rate and predicted survival rate were almost in agreement by observing the calibration curve (Figure 4B). It validated the high accuracy of the nomogram in predicting the survival rate of gastric cancer patients. In addition, we also found the largest area under the ROC curve for the nomogram (AUC = 0.740) (Figure 4C). It implied that the nomogram predicted patients’ survival better than other clinical characteristics. The nomogram was confirmed to be an indicator of independent prognosis by the results of univariate and multivariate Cox analyses (Figures 4D,E). We randomly selected four prognostic signature articles of gastric cancer in the latest 3 years from the PubMed website (https://pubmed.ncbi.nlm.nih.gov/), including Dai’s signature (ITGAV, DAB2, SERPINE1, MATN3, and PLOD2), Liu’s signature (NOX4, NOX5, GLS2, MYB, TGFBR1, NF2, AIFM2, ZFP36, SLC1A4 TXNIP, CXCL2, HAMP, and SP1), Meng’s signature (CGB5, IGFBP1, OLFML2B, RAI14, SERPINE1, IQSEC2, and MPND), and Yin’s signature (GPX3, ABCA1, NNMT, NOS3, SLCO4A1, ADH4, DHRS7, and TAP1) (Meng et al., 2020; Liu SJ et al., 2021; Dai et al., 2021; Yin et al., 2021). To highlight the advantages of the cellular senescence signature, we compared these five signatures, and the results are visualized in Supplementary Figure S5. We found that the cellular senescence signature was the best predictor of prognosis in gastric cancer patients, with a C-index of 0.642.

### Risk Score Guide Clinical Treatment

**FIGURE 5:**
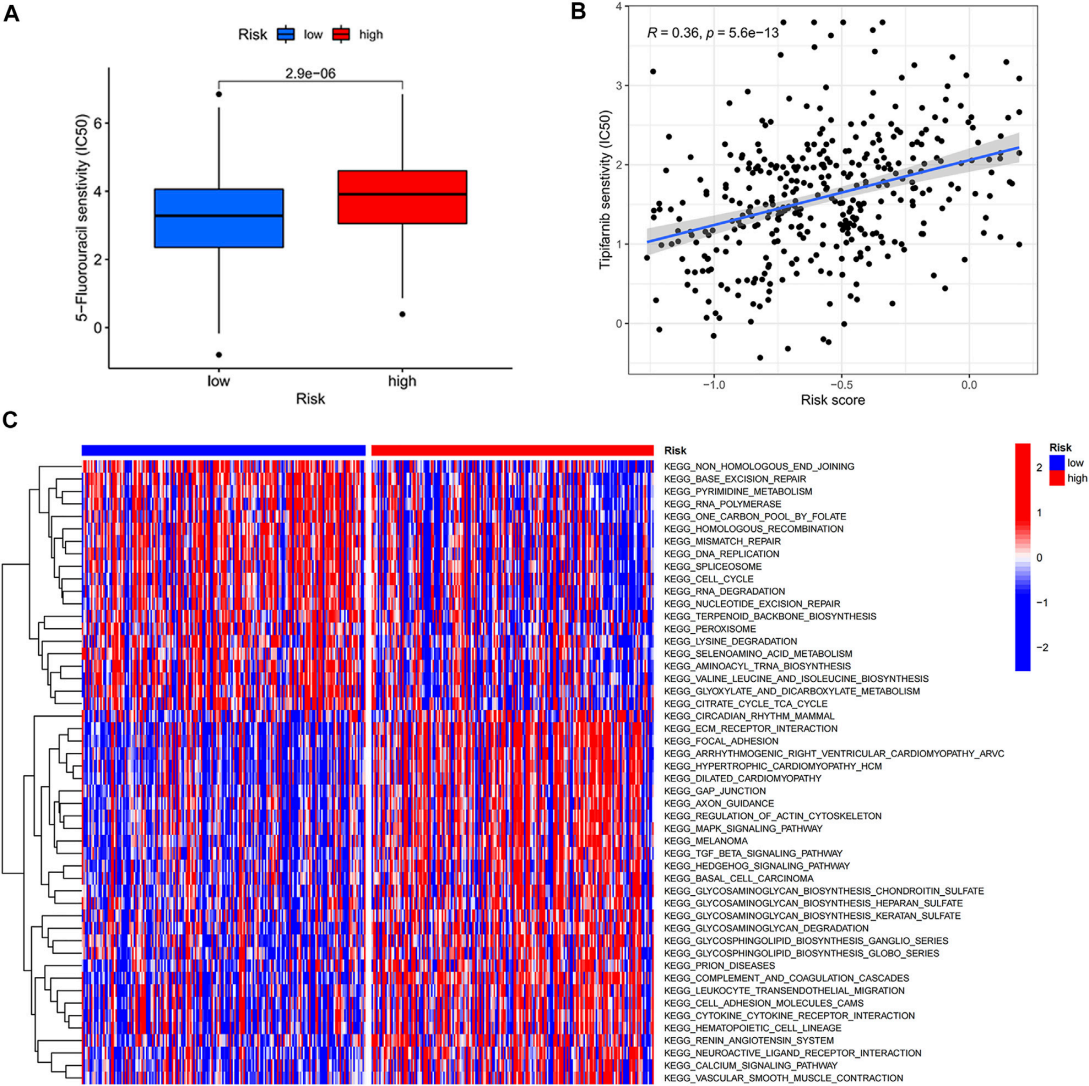
Risk score-guided chemotherapy. **(A)** Box plot of IC_50_ differential analysis for high- and low-risk score groups. **(B)** Scatter plot of correlation between risk score and IC_50_. **(C)** Heat map of the differential analysis of GSVA enrichment between high- and low-risk groups.

**FIGURE 6:**
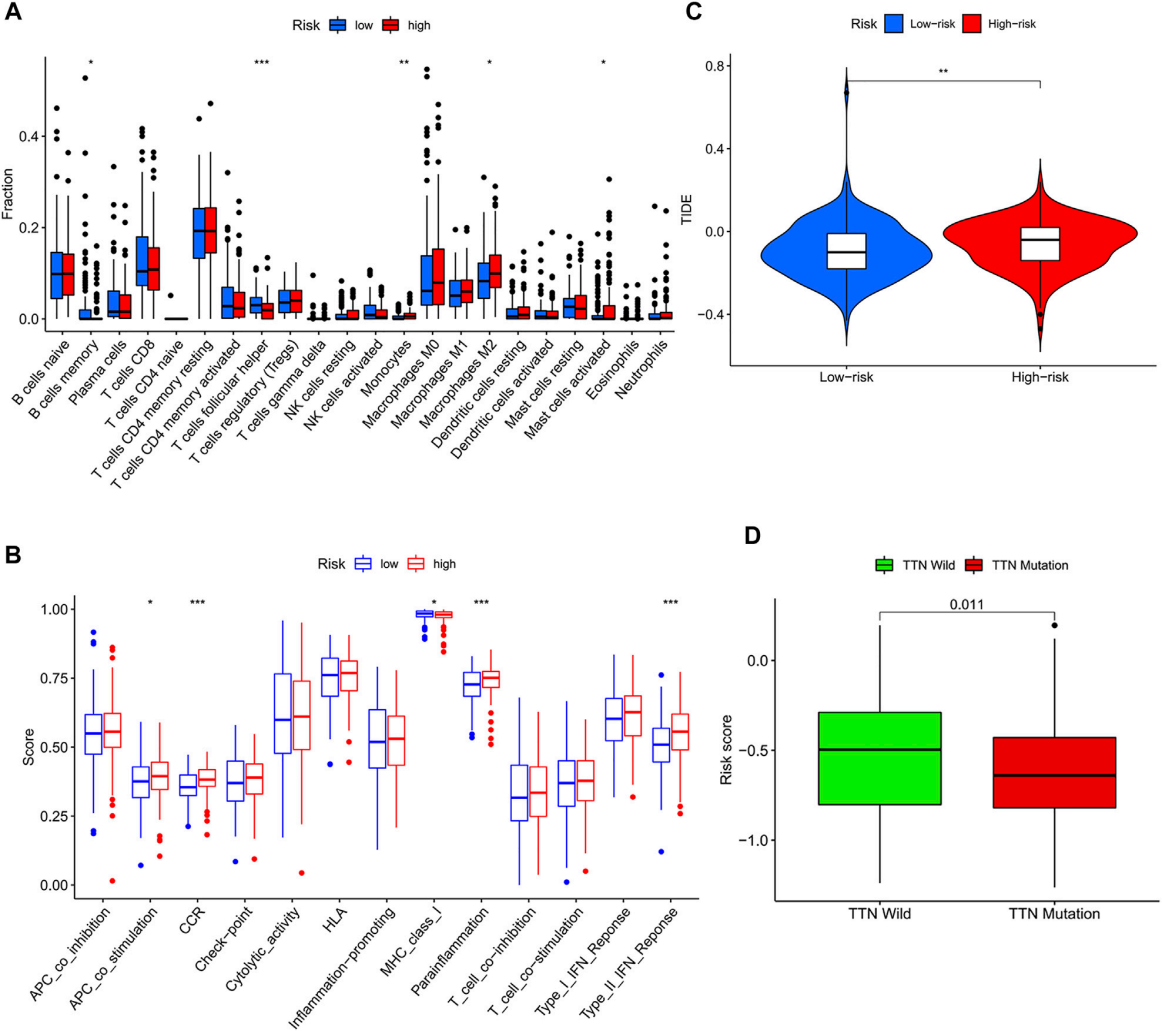
Risk score-guided immunotherapy. **(A)** Box plot of the differential analysis of immune infiltration between the two risk score groups. **(B)** Box plot of the differential analysis of immune function between two risk score groups. **(C)** Violin plot of the response to immunotherapy between the two risk fraction groups calculated by the IDE algorithm. **(D)** Box plot of the differential analysis of the risk score between wild type and mutant type for the top 10 mutated genes in TCGA data.

Due to the increase in tumor resistance to chemotherapeutic drugs, most patients with gastric cancer currently have poor chemotherapy outcomes. We explored whether risk scores could play a role in chemotherapy. In our study, the risk score was significantly and positively correlated with half-maximal inhibitory concentration (IC_50_), and the low-risk score group had a lower IC_50_ value and was more sensitive to 5-FU (Figures 5A,B). By performing GSVA, most of the senescence pathways were found to be more active in the low-risk score group (Figure 5C). The high-risk score group had higher macrophage M2 infiltration, and the low-risk score group had higher B-cell memory and T-cell follicular helper infiltration (Figure 6A). In addition, immune function analysis showed that type_II_IFN_response and parainflammation were more active in the high-risk group, and MHC_class_I was more active in the low-risk group (Figure 6B). It suggested that the low-risk group might be more suitable for immunotherapy. It was confirmed by the TIDE algorithm that patients in the low-risk score group are more suitable for immunotherapy (Figure 6C). The risk score signature constructed using cellular senescence-related genes is a potential biomarker for assessing the clinical response to immunotherapy in gastric cancer patients. We also identified the top 10 mutated genes (TTN, TP53, MUC16, ARID1A, LRP1B, SYNE1, FLG, FAT4, CSMD3, and PCLO), SMARCA4, and ZFP36 in TCGA data (Supplementary Table S4). The samples were classified into mutation and wild types according to the mutation status of the genes. Among them, the mutation type of the six genes (TTN, ARID1A, LRP1B, FLG, FAT4, and PCLO) had lower risk scores (Figure 6D). We also found no difference in risk scores between mutation and wild type of SMARCA4 and ZFP36 (Supplementary Figure S6), so we speculated that co-mutation of SMARCA4 and ZFP36 does not affect the prognosis of gastric cancer.

### Differential Gene Enrichment Analysis

**FIGURE 7:**
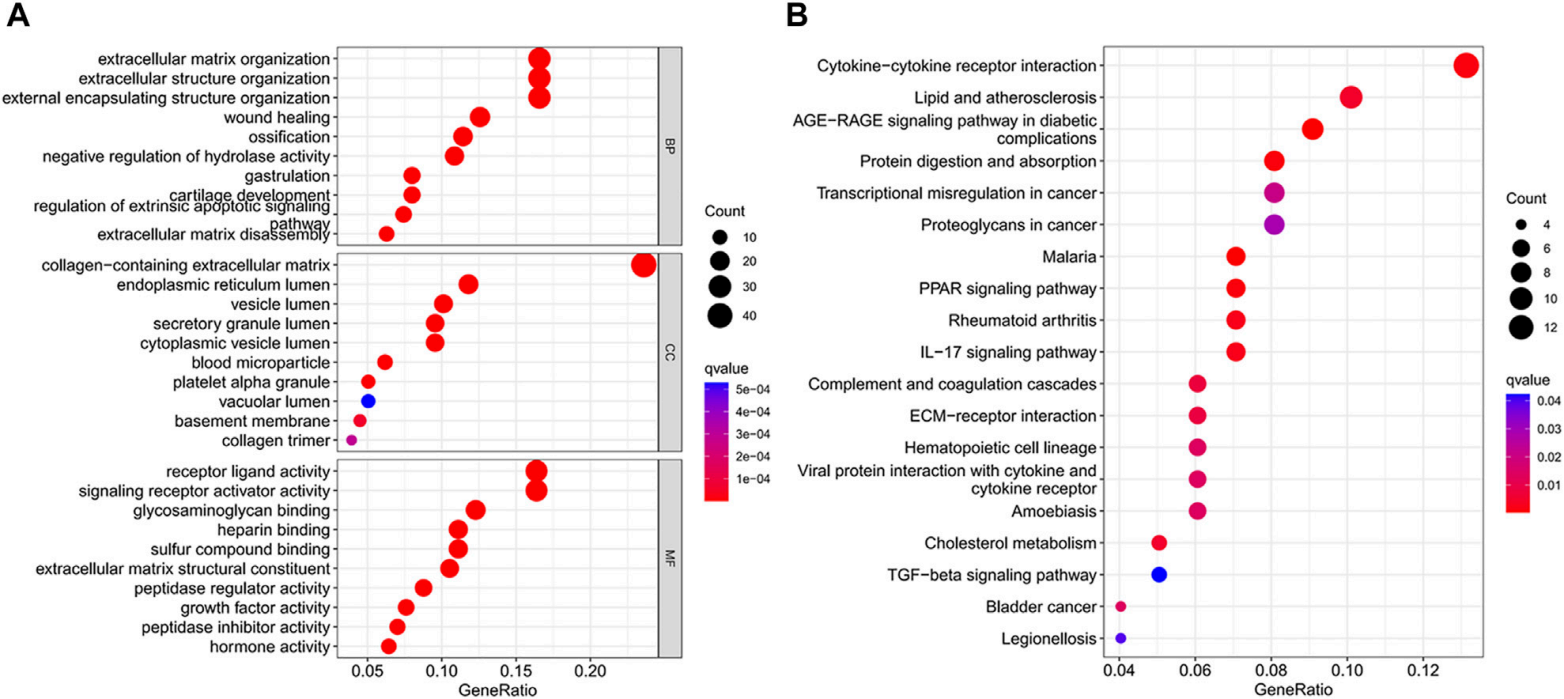
Differential gene enrichment analysis. **(A)** GO enrichment analysis bubble map. **(B)** Bubble plot of KEGG enrichment analysis.

We identified 186 differential genes in two risk groups. GO and KEGG enrichment analyses were performed on the differential genes, and the enrichment results were visualized in bubble plots. We found that extracellular matrix organization, extracellular structure organization, and external encapsulating structure organization were significantly enriched in the GO bubble map (Figure 7A), while cytokine–cytokine receptor interaction, protein digestion and absorption, transcriptional misregulation in cancer, and proteoglycans in cancer were significantly enriched in the KEGG bubble plots (Figure 7B).

### Identification of 10 Hub Genes

**FIGURE 8:**
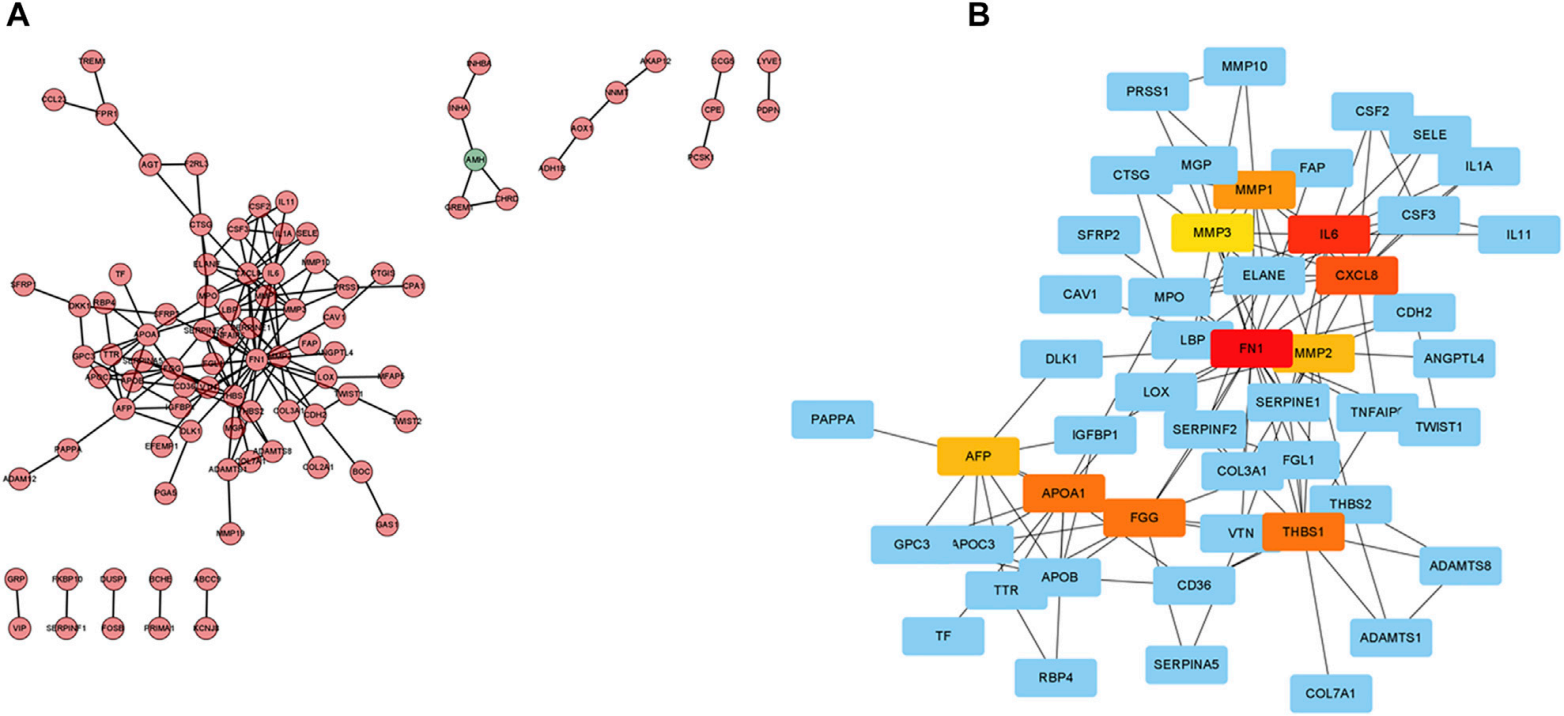
Protein–protein interaction (PPI) network. **(A)** Cytoscape-treated PPI network. Red, highly expressed DEGs in the high-risk score group; green, highly expressed DEGs in the low-risk score group. **(B)** CytoHubba identified the top 10 hub genes.

The expression profiles of DEGs in two risk score groups were evaluated using the STRING database. PPI networks were constructed (Supplementary Figure S7). By using Cytoscape software, PPI network data were processed and displayed. The interactions of DEGs are shown in Figure 8A. A total of 10 hub genes (FN1, IL6, CXCL8, THBS1, APOA1, FGG, MMP1, AFP, MMP2, and MMP3) of DEGs were identified using Cytoscape plugin cytoHubba and the extent method (Figure 8B). A total of six upregulated genes (FN1, APOA1, CXCL8, MMP1, MMP3, and THBS1) in tumor tissue were identified by the differential analysis of 10 hub genes (Supplementary Figure S8). By further analysis, five hub genes (FN1, APOA1, CXCL8, MMP1, and THBS1) with survival differences were identified (Supplementary Figure S9). Patients with low-expression levels had a better prognosis. We also analyzed the differences in the expression levels of genes in different clinical characteristics. FN1 was significantly more expressed in patients after stage T1 and unchanged in patients after stage T2. The expression levels of FN1 and THBS1 were higher in G3 patients than those in G2 patients (Supplementary Figures S10, 14). The expression levels of APOA1 were higher in G2 patients than those in G3 and in N2 than those in N0 (Supplementary Figure S11). The expression level of CXCL8 was significantly higher in patients over 65 years of age and after stage III (Supplementary Figure S12). The expression level of MMP1 was significantly higher in patients over 65 years of age and in stage IV than that in stage I (Supplementary Figure S13). Finally, we also performed the differential analysis of immune cell infiltration (Supplementary Figure S15). The FN1, APOA1, CXCL8, MMP1, and THBS1 low-expression groups all had higher immune cell infiltration and might be suitable for immunotherapy.

## Discussion

Cellular senescence is the result of irreversible cessation of cell division (Gorgoulis et al., 2019). Studies have shown that it can occur in the context of oncogene activation and is involved in tumor suppression (Di Micco et al., 2021). The latest studies have shown that senescent cancer cells have not only antitumor activity but also pro-tumor activity. Cellular senescence can play an essential role in immune surveillance to ensure that senescent cancer cells are eliminated (Prasanna et al., 2021). Nowadays, cellular senescence is emerging as a potentially novel anticancer strategy (Ramu et al., 2021). It could help guide effective anticancer therapy strategies by exploring the cellular senescence patterns of GC.

The main purpose of this study was to discuss the effect of cellular senescence on the prognosis and treatment of GC. We constructed a prognostic risk score signature for cellular senescence-related genes using TCGA data. Patients with low-risk scores had longer survival times, while the opposite was true for patients with high-risk scores. The same results were found in the GEO data. It indicated that the prognostic risk score signature could forecast the GC patients’ prognosis. We also observed that the prognostic risk score signature could be an independent prognostic factor for GC by further Cox analysis. In addition, a nomogram was constructed for predicting gastric cancer patients’ survival. The calibration curve confirmed the predictive accuracy of the nomogram. Encouragingly, the area under the ROC curve (AUC) of the nomogram was significantly higher than other clinical features, especially in traditional TNM stages. It showed that the nomogram has higher accuracy in predicting 1-year, 3-year, and 5-year survival rates of gastric cancer patients than clinical TNM stages. Moreover, the cellular senescence signature had the highest C-index and predicted the best prognosis among other prognosis-related signatures of gastric cancer.

Although chemotherapeutic agents are helpful in the therapy of GC, many GC patients appear resistant to chemotherapy, resulting in poorer chemotherapy outcomes (Wei et al., 2020). Therefore, it is increasingly essential to identify GC patients who are sensitive to chemotherapeutic drugs. According to these reasons, we investigated the differences in clinical response to chemotherapeutic drugs in two risk groups. In our research, GC patients with low-risk scores were more susceptible to 5-FU. It suggested that using the risk score could identify gastric cancer patients who are more suitable for chemotherapy. With the development of technology, more and more therapeutic approaches are available for GC (Hsu and Raufi, 2021). Immunotherapy is an emerging cancer treatment that activates the body’s immune system to clear tumor cells (Kawazoe et al., 2021). The identification of patients with gastric cancer suitable for immunotherapy is particularly critical in the clinical environment. We observed higher immune infiltration levels in the low-risk score group, including B-cell memory and T-cell follicular helpers, and the high-risk score group had higher infiltration levels of macrophage M2 (tumor-promoting cells) (Xia et al., 2020; Overacre-Delgoffe et al., 2021). The results of immune function analysis also showed that high-risk score patients had active immune-related functions “type_II_IFN_response” and “parainflammation,” whereas “MHC_class_I” was more active in the low-risk score group. Previous studies have shown that “type_II_IFN_response” is considered an anticancer immune-related function (Liu M et al., 2020). Interestingly, our findings showed the opposite that “type_II_IFN_response” might promote the development of gastric cancer. The immune-related function “parainflammation” is thought to promote tumor progression, which is consistent with our findings (Aran et al., 2016). The immune-related function “MHC_class_I,” which mainly plays a role in the immunosurveillance of cancer, inhibits the immune escape of tumors and is considered a potential target for cancer immunotherapy (Cornel et al., 2020; Dersh et al., 2021). We speculated that patients with low-risk scores may be suitable for immunotherapy. Next, we demonstrated that low-risk score patients had a low immune escape potential and were more sensitive to immunotherapy using the TIDE algorithm. In conclusion, the prognostic risk score signature with cellular senescence genes not only predicts prognosis but also identifies patients with chemotherapy- and immunotherapy-sensitive gastric cancer. We also analyzed the top 10 mutated genes in the TCGA data. The mutation types of TTN, ARID1A, LRP1B, FLG, FAT4, and PCLO had lower risk scores than the wild type. This meant that patients with mutation types might have a better prognosis and be more suitable for chemotherapy and immunotherapy. We also identified a particular gene SMARCA4. It has the highest mutation frequency (6%), and it is linked to higher overall survival (OS) and progression-free survival (PFS). There was a co-mutation relationship between SMARCA4 and ZFP36. But we found no difference between mutation and wild type in SMARCA4 and ZFP36, so we speculated that co-mutation of SMARCA4 and ZFP36 does not affect the prognosis of gastric cancer.

Because of the significant differences between the two risk groups, it is essential to study the differential genes in depth. We identified 10 hub genes (FN1, IL6, CXCL8, THBS1, APOA1, FGG, MMP1, AFP, MMP2, and MMP3) by constructing a PPI network. FN1, APOA1, CXCL8, MMP1, MMP3, and THBS1 were significantly upregulated in the tumor samples. This result was confirmed in the HPA database. We observed that FN1, APOA1, CXCL8, MMP1, and THBS1 were correlated with GC prognosis, with higher expression levels associated with a worse prognosis. This is consistent with previously published research studies (Li et al., 2019; Chen X et al., 2020; Chen YJ et al., 2020; Liu X et al., 2020; Zhang et al., 2021). We also found higher immune infiltration (plasma cells and T cells) in the low-expression group of FN1, APOA1, CXCL8, MMP1, and THBS1, while macrophages M2 and resting T cells showed higher infiltration in the high expression group. It suggested that patients in the low-expression group of FN1, APOA1, CXCL8, MMP1, and THBS1 might be more suitable for immunotherapy.

In summary, the cellular senescence risk score prognostic signature could be used to assess the prognosis of GC patients and guide clinical treatment. Our study not only provided a new predictive signature for the prognosis of GC but also offered guidance for the future therapy of gastric cancer.

## Data Availability

Publicly available datasets were analyzed in this study. The names of the repository/repositories and accession number(s) can be found in the article/Supplementary Material.
