# Supplementary material for: Cellular Senescence-Related Genes: Predicting Prognosis in Gastric Cancer
Source: Front Genet. 2022 Jun 1;13:909546. doi: 10.3389/fgene.2022.909546 (PMC9198368; doi:10.3389/fgene.2022.909546)
Supplement: Supplementary file 3 [file Table1.DOCX]

1. RNA sequencing (RNA-Seq), clinical data, and mutation data of STAD were downloaded from the The Cancer Genome Atlas (TCGA):

<https://portal.gdc.cancer.gov/>. Microarray data from Affymetrix were obtained from GEO (<https://www.ncbi.nlm.nih.gov/geo/> , GSE84437). Cellular senescence-related genes were downloaded from CellAge (<https://genomics.senescence.info/cells/> ).

1. TIDE algorithm was acquired from <http://tide.dfci.harvard.edu/> .
2. The human genome annotation data downloaded from the GENCODE website (<https://www.gencodegenes.org/human/> ).
3. R. software: <https://www.r-project.org/> .
4. We used the R package pRRophetic to evaluate their therapy response determined by the half-maximal inhibitory concentration (IC50) of each GC patient on Genomics of Drug Sensitivity in Cancer (GDSC) (<https://www.cancerrxgene.org/> ).
5. The STRING database: <https://string-db.org/> .
6. Cytoscape software: <https://cytoscape.org/> .
