# Supplementary material for: Cellular Senescence-Related Genes: Predicting Prognosis in Gastric Cancer
Source: Front Genet. 2022 Jun 1;13:909546. doi: 10.3389/fgene.2022.909546 (PMC9198368; doi:10.3389/fgene.2022.909546)
Supplement: Supplementary file 6 [file Table6.DOCX]

<https://www.jianguoyun.com/c/sd/14d9b67/2247b32cb406962e>
